# Multicolour Emission States from Charge Transfer between Carbon Dots and Surface Molecules

**DOI:** 10.3390/ma10020165

**Published:** 2017-02-11

**Authors:** Shengliang Hu, Yanbing Wang, Wenyu Zhang, Qing Chang, Jinlong Yang

**Affiliations:** 1 School of Material Science and Engineering, North University of China, Taiyuan 030051, China; wybingzbdx@yeah.net (Y.W.); zwyu2017@163.com (W.Z.), jinlongnuc@yeah.net (J.Y.); 2 State Key Laboratory of New Ceramics and Fine Processing, Tsinghua University, Beijing 100084, China

**Keywords:** carbon dots, band gap, surface states, charge transfer, photoluminescence, heteroatom groups

## Abstract

The emissive states of carbon dots have been tuned by controlling the charge transfer process. The carbon dots couple with molecules, which are made of a benzene ring and different heteroatom substituents, through amino-carboxylic bonds that are generally identified as charge transfer promoters at the interface. New ways of radiative recombination are created due to the transfer of photo-excited electrons from carbon dots to the lowest unoccupied molecular orbital (LUMO) of the grafted molecules. By variation of the molecular orbital energy levels via heteroatom substituents in the benzene ring, the different optical properties and emission colors of the carbon dots were presented. This work opens up new opportunities for the application of carbon dots since different heteroatom substituents could lead to many possibilities for conjugation with drugs and biomolecules.

## 1. Introduction

Carbon dots (CDs), a class of zero-dimensional carbongenic nanomaterials with bright fluorescence, have attracted considerable attention because of their elemental nontoxicity, facile synthesis, and their potential applications in imaging, sensing, lighting, catalysis, and photovoltaics [1,2,3,4,5]. The photoluminescence (PL) from CDs are highly sensitive to a number of factors [6]. For the CDs resembling the crystalline structure of single or a-few layered graphene (called graphene quantum dots, GQDs), the theoretical modelling and calculations suggest that their energy gaps can be tuned by the lateral size, shape, edge configuration, and thickness [7,8,9,10]. However, completely consistent results are difficult to be observed in experiments [6]. This could be caused by the huge heterogeneity of individual particles from the preparation sample. In addition, the theoretical calculations are generally limited without taking all factors into account simultaneously [8,10]. 

For quasi-spherical CDs, many works have demonstrated that their PL tuning depends more on surface states instead of sp^2^ clusters in the core, though the quantum confinement effect also plays a role [4,6,11,12]. Such CDs have ultrahigh specific surface areas that enables their surface-facet atoms to be as important as those on the inside. Therefore, surface incorporation methodologies such as heteroatom incorporation and surface functionalization have become effective ways to tune the electronic and opto-electronic properties of CDs [11,13,14,15,16,17]. Recent studies have demonstrated the key roles that O and N elements or their related groups play in engineering band gaps of CDs when they are incorporated into their external lattice framework [17,18,19,20,21,22,23,24]. Other elements such as S and P could enhance the effects of O and N-contained groups on the band gaps in CDs through a cooperative effect [25,26,27,28,29,30]. Multi-color light-emitting CDs were obtained by tuning O and/or N concentrations and their bonding types with C in CDs [12,18,19,20,22,23,24,31,32,33,34]. In principle, it is generally accepted that the incorporation of heteroatoms in the carbon lattice disrupts sp^2^ hybridization of carbon atoms and thereby results in the formation of new band gaps in CDs [12,18,20,22,34]. 

Besides the above mechanism of tailoring the band structure by heteroatom-induced defect states and energy traps, charge transfer, which is a basic process governing the electronic and opto-electronic properties of a material, can be further used to tune emissive sites in CDs [15,35,36]. Sun’s group has firstly demonstrated the ability of CDs to transfer their surface-confined electrons and holes [37]. Then Jin et al. provided the experimental and theoretical evidence in support of charge-transfer-controlled electron-hole recombination [35]. They found the electron transfer from amine groups to six-member carbon rings, causing the variation of band gaps in CDs. However, the change of band gaps is still limited for CDs by attaching amine groups. Accordingly, developing a molecule implantation scenario for specially tuning emissive sites in CDs through the charge transfer effect is essential not only for the application of CDs but also for understanding their interaction with surface-attached molecules.

Here we present a new approach to tuning emissive states in CDs by grafting molecules consisting of a benzene ring and different heteroatom groups on the surface of CDs through amino-carboxylic bonds. In this system, the amino-carboxylic molecular junction acts as a conduit for electron transfer between the CDs and the surface-attached molecules, while the heteroatom groups bonding with the benzene ring are employed to regulate the molecular orbital energy levels.

## 2. Experimental Section

**Figure 1:**
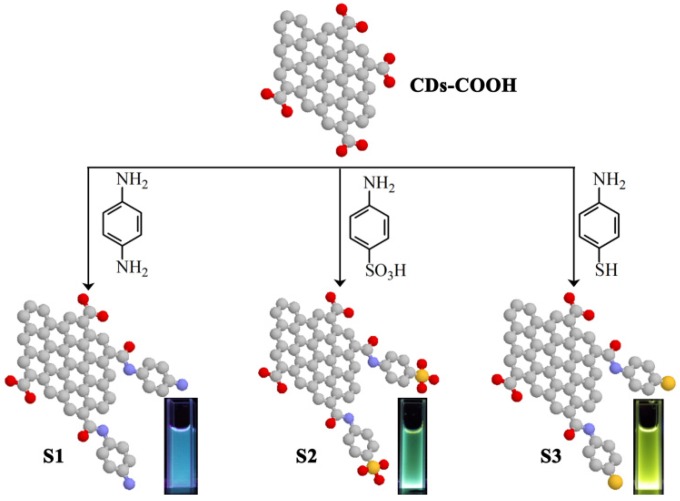
Schematic of modifying CDs with p-phenylenediamine (S1), sulfanilic acid (S2), and 4-aminothiophenol (S3), respectively, and their corresponding emission photos under UV excitation of 356 nm.

CDs with abundant –COOH groups, named as CDs-COOH, were firstly fabricated by solvothermally treating citric acids [38]. Subsequently they were modified with p-phenylenediamine (S1), sulfanilic acid (S2), and 4-aminothiophenol (S3), respectively, through acylation reaction forming amino-carboxylic bonds (Figure 1).

Preparation of CDs-COOH followed procedures reported previously [38]. Briefly, 1.68 g of citric acid was dissolved in 40 mL of DI water. The obtained solution was transferred to a Teflon-lined autoclave (100 mL) and then heated at 200 °C for 5 h. After cooling to room temperature naturally, the transparent solution containing CDs-COOH was obtained by 30 min centrifugation (10,000 rpm). 0.25 mmol of p-phenylenediamine, sulfanilic acid, or 4-aminothiophenol were dissolved into the same volume of CDs-COOH solution (10 mL), respectively. Then the solution was put into a 50 mL Teflon-lined autoclave and was heated at 135 °C for 5 h again. Thus, the molecule-modified CDs, i.e., S1, S2, and S3, respectively, were obtained after cooling to room temperature. To compare, the samples were also prepared by changing the amounts (e.g., 0.125, 0.375 mmol) of the p-phenylenediamine, sulfanilic acid, or 4-aminothiophenol under the same conditions. Moreover, the samples were also prepared by the same process with reaction temperature variation (e.g., 70, 90, 160, 200 °C). All sample solutions were adjusted to the same pH value by neutralization with base.

All photoluminescence (PL) spectra were obtained by a fluorescence spectrophotometer (Hitachi, Tokyo, Japan). X-ray photoelectron spectroscopy (XPS) data of all samples was collected by a Kratos AXIS 165 multitechnique electron spectrometer (Shimadzu, Tokyo, Japan) with an Al Kα X-ray source for determining the composition and chemical bonding configurations. Transmission electron microscopy (TEM) measurements were performed on a Tecnai G2 F20 electronic microscope (FEI, Fremont, CA, USA). UV–Vis absorption was characterized by a UV-2550 UV-vis spectrophotometer (Shimadzu, Tokyo, Japan). The time-resolved PL spectra were obtained on an FLS 920 PL spectrometer (Edinburgh Instruments, Livingston, UK). The infrared spectra were collected on a Thermo Nicolet 360 FT-IR spectrophotometer (Thermo Fisher Scientific, Waltham, MA, USA).

## 3. Results and Discussion

As shown in Figure 1, three kinds of heteroatom groups, i.e., –NH_2_, –SO_3_H, and –SH, are also introduced in CDs with molecule implantations. Under the 365 nm UV lamp, the markedly different emission colors were observed from the obtained samples of S1, S2 and S3, respectively.

**Figure 2:**
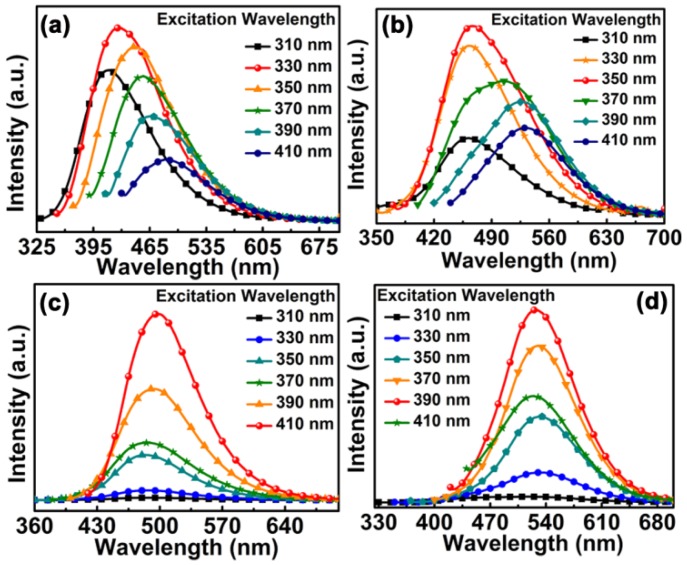
PL spectra of (**a**) CDs-COOH, (**b**) S1, (**c**) S2, and (**d**) S3 excited with different wavelengths.

Figure 2 shows the PL spectra of CDs-COOH, S1, S2, and S3 excited with the different light wavelengths. For each sample of CDs, there were one strongest PL peak and one optimal excitation wavelength, reflecting the most dominant emissive sites in the CDs. The strongest emission peaks of CDs-COOH, S1, S2, and S3 are around 425, 465, 500, and 530 nm, respectively. A striking shift in PL emission with varying excitation is observed in CDs-COOH and S1 (Figure 2a,b). However, there is an obvious difference in the PL spectra other than the PL peak variations. The redshift degree for CDs-COOH is proportional to the increase of the excitation wavelength, whilst the PL peaks of S1 do not change obviously when the excitation wavelength is smaller than the optimal wavelength. Both S2 and S3 reveal excitation-wavelength-independent PL properties, but their optimal excitation wavelengths are still different (Figure 2c,d). In order to offer insight into these differences of PL behaviors presented in Figure 2, we performed further characterizations of sizes and compositions, which are possible factors for tuning the band gaps in CDs [12].

**Figure 3:**
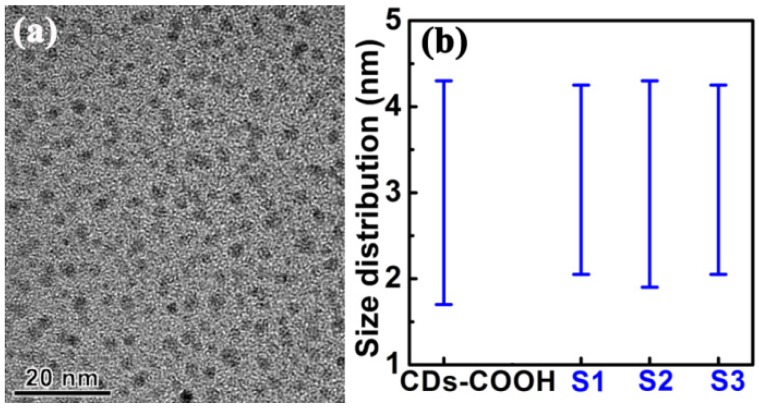
A typical TEM image of (**a**) CDs and (**b**) the size distributions of CDs-COOH, S1, S2, and S3.

The TEM images reveal that CDs from the samples of CDs-COOH, S1, S2, and S3 are well dispersed (Figure 3a) and exhibit similar size distribution (Figure 3b). Moreover, the lattice fringes of CDs show the same interplanar spacing on the basis of their high resolution TEM (HRTEM) images (Figure S1). Although the concentration of CDs could also modulate their PL centers according to the reports of Yu’s group [39], this case was not observed in this work. Accordingly, the differences in PL from CDs-COOH, S1, S2, and S3 could be independent of their sizes.

**Figure 4:**
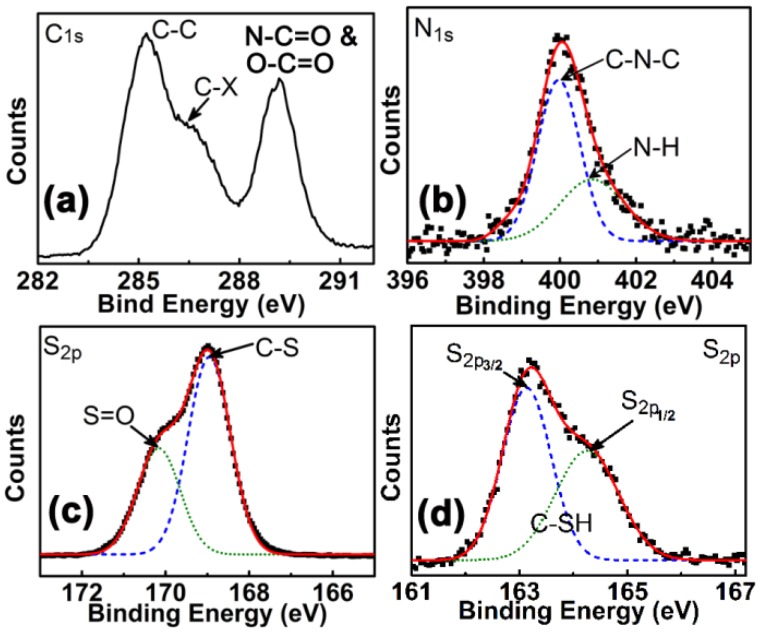
XPS spectra for modified CDs (**a**) a typical C1s; (**b**) N1s; (**c**) S2p of S2 and (**d**) S2p of S3.

The surface compositions of CDs were determined by XPS, which has become a main tool that is widely used to characterize these materials. A similar C1s signal is observed from the CDs-COOH, S1, S2, and S3 samples, as shown in Figure 4a. There are clearly three different chemical environments, corresponding to C–C, C-X (X=O, N, or S), and N–C=O/O-C=O bonds, respectively [12,20,23,26,28]. The N element is presented in all the CDs from S1, S2, and S3 due to molecular modification (Figure 4b). Their N1s XPS spectra are nearly identical and can be resolved into two peaks at 399.7 and 401.2 eV, which are attributed to the C–N–C (pyrrolic N) and N–H bonds, respectively [28,29,31]. This also implies the presence of amino-carboxylic bonds in S1, S2, and S3 after molecule modification. Besides the signals of C, N, and O, the S2p peaks at different binding energies are observed in CDs from S2 and S3, respectively (Figure 4c,d). According to the high resolution scan of S2p, the bonds of C–S and S=O are present in the CDs from S2, while the S2p_3/2_ and S2p_1/2_ of C–S–H bonding exist in those from S3 [25,26,27,28], suggesting that there are different S-contained groups in the CDs from S2 and S3. Infrared (IR) spectra (Figure S2) confirmed the presence of vibrational absorption bands of C=O from CONH, and C–N in CDs modified by molecules, and revealed the stretching peaks of the –SO_3_H and –SH groups in the CDs from S2 and S3, respectively. The above results clearly demonstrate that the molecules of p-phenylenediamine, sulfanilic acid, and 4-aminothiophenol are grafted on CDs through amino-carboxylic bonds and then give rise to the changes of their chemical structures and compositions. This could result in the PL tuning of CDs.

Previous works have postulated that the low PL efficiency of only *O*-incorporated CDs is not only highly associated with many kinds of possible recombination, but is attributed to the charge transfer induced by C=O bonds [40,41]. Consequently, both heteroatom incorporation and molecule implantation are always used for suppressing nonradiative recombination and charge transfer to improve the PL of CDs [2,13,25,31]. For instance, Li et al. demonstrated that the absorption and PL of CDs were modified after they were conjugating with polyethylene glycol (PEG) polymers of different molecular weights via amino-carboxylic bonds [42]. According to the work reported by Kladnik et al., however, the amino-carboxylic bonds connecting CDs with the surface molecules can serve as a fast tunnel of charge transfer [43]. Why do the CDs after molecular modification exhibit brighter luminescence than the CDs-COOH in the present work? This can be ascribed to the benzene ring in the molecule structure employed, which is an antibonding state and responsible for reserving electrons or energy [35]. Thus, the possibility that the excitation energy returns to the ground state by emitting a photon rather than being lost as heat is enhanced.

**Figure 5:**
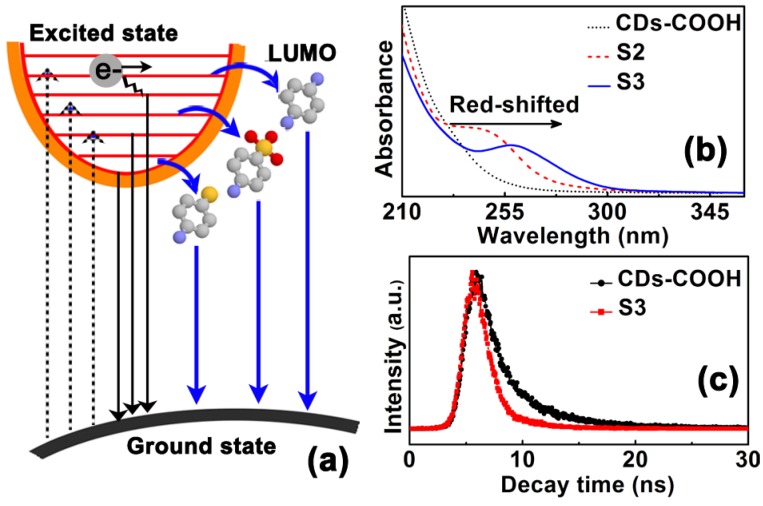
(**a**) Illustration for the PL mechanism of modified CDs; (**b**) Light absorption comparisons of CDs-COOH, S2, and S3; (**c**) PL decay profiles of CDs-COOH and S3 excited by the same wavelength.

It has been confirmed that a series of electronically excited states can be created in CDs with O incorporation [20,23]. When the energy levels of these excited states are higher than those of the molecular orbital of the molecules grafted on the surface of the CDs, the electrons excited in CDs will be quickly transferred to their lowest unoccupied molecular orbital (LUMO) through the amino-carboxylic molecular junction as shown in Figure 5a. Because the electron density in the benzene ring highly depends on its substituent groups [35], their changes will significantly influence the LUMO levels of superficial molecules and then cause band gap shifts of molecule-modified CDs. For instance, the electron density in the benzene ring can be reduced by the SO_3_H groups as an electron-withdrawing substituent; whereas it can be enhanced by the SH groups possessing strong electron-donating abilities. From Figure 5b, a typical new absorption band at around 250 nm, which is attributed to the benzene ring, is presented after the molecular modifications of CDs-COOH. Remarkably, a redshift of this absorption band can be observed by comparing S2 with S3, due to the increased electron density in benzene ring. To further verify this, S3 was treated with HNO_3_, thus removing the SH groups from the CDs in S3. The treated sample yields blue shifts greater than 30 nm in the PL emission when compared with S3 (Figure S3).

As mentioned above, the amino-carboxylic molecular junction is an ultrafast electron transfer pathway [43], and consequently, the migration of excitation energy from the CDs to their superficial molecules is predominant due to the absence of long-time trips [36]. As shown in Figure 5c, PL decay studies reveal a markedly shortened lifetime of S3 (2.32 ns) in contrast with that of CD-COOH (5.40 ns), confirming the suppression of energy loss to the hole trapping and structural defects. Recently, some works also reported S-doped CDs, in which a variety of S-contained groups coexisted [25,26,27,28]. However, the PL emission of these S-doped CDs mainly exhibited blue or cyan colors and did not rely on the types of S-contained groups. To clarify whether the PL shifts between S2 and S3 are related to S contents in CDs or not, control experiments were conducted. During the surface modification for CDs-COOH, not only the reaction temperature but also the amount of added molecules such as sulfanilic acid and 4-aminothiophenol were changed. Although these factors could lead to large changes of the PL intensity, but they did not cause obvious shifts in the peak PL emission (Figures S4 and S5). Thereby, the charge transfers rather than the inter-band crossings are dominant in radiative recombination for molecule-modified CDs.

## 4. Conclusions

Molecules containing a benzene ring and different heteroatom substituents were employed to modify CDs through the acylation reaction, forming amino-carboxylic bonds. The different PL behaviors and emission colors were presented with the variations of heteroatom substituents in the used molecules. This could be attributed to photo-excited electron transfer from CDs to different LUMO levels of modified molecules via amino-carboxylic molecular junctions. Not only does this work provide a novel approach for tuning the emissive states in CDs and could be beneficial in understanding the rules governing the charge transfer from CDs to their surfaces, but it also enables new opportunities in the use of CDs, since different heteroatom groups could lead to many probabilities for conjugation with ions and molecules. For instance, the CDs from S2 can be used for the selective detection of Fe^3+^ in live cells and aqueous solutions (Figure S6) owing to the introduction of SO_3_H groups. Our presented CDs are also expected to expand the choices for drug delivery, tracers, biosensing, and so on through the selective combination of heteroatom groups in CDs with drugs or organic molecules.

## Supplementary Materials

The following are available online at www.mdpi.com/1996-1944/10/2/165/s1, Figures S1–S6.
