# Supplementary material for: Multicolour Emission States from Charge Transfer between Carbon Dots and Surface Molecules
Source: Materials (Basel). 2017 Feb 11;10(2):165. doi: 10.3390/ma10020165 (PMC5459146; doi:10.3390/ma10020165)
Supplement: Supplementary file 1 [file materials-10-00165-s001.pdf]

Supplementary Materials

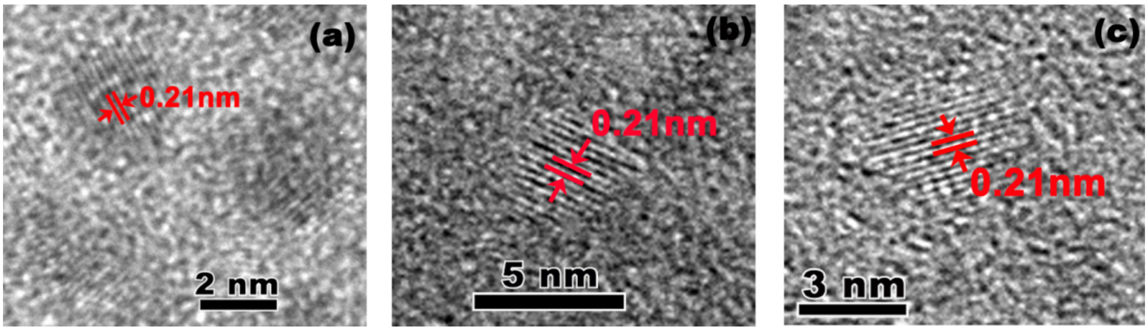

Figure S1. HRTEM images of molecule-modified CDs from S1 (**a**); S2 (**b**) and S3 (**c**), respectively.

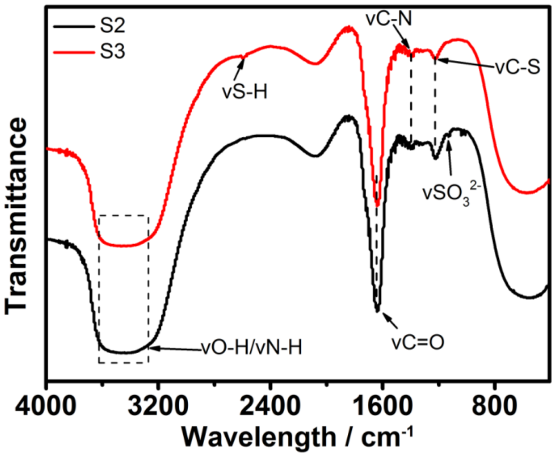

Figure S2. IR spectra of S2 and S3.

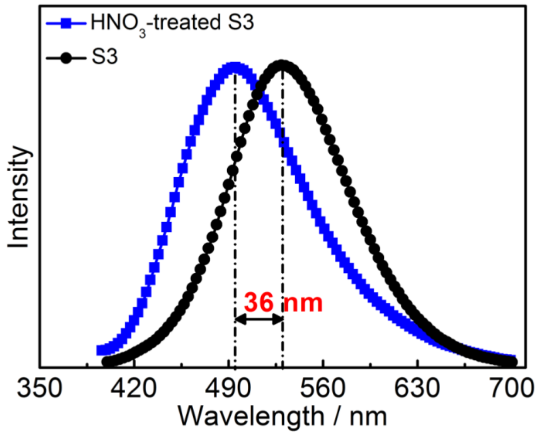

Figure S3. PL spectra of S3 and HNO_3-treated S3. Here, 2.5 mL of concentrated HNO_3 was added in 10 mL of S3 solution. Then the mixed solution heated at 40–50 °C for 3 h under the stirring. The obtained solution was neutralized with base and then removed the excess salt and water to keep the carbon dot concentration unchanged.

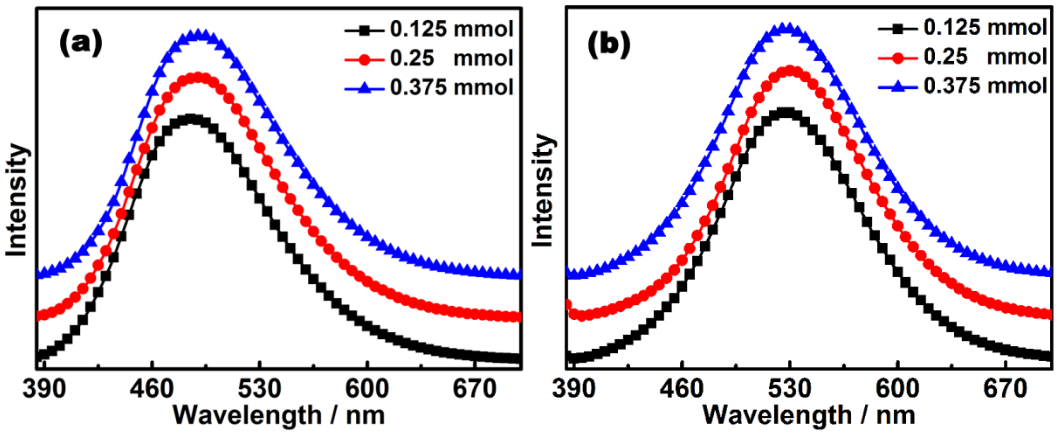

Figure S4. PL spectra of the samples obtained by changing the amounts of sulfanilic acid (**a**) and 4-aminothiophenol (**b**) adding into the 10 mL CDs-COOH solution at the same excitation wavelength.

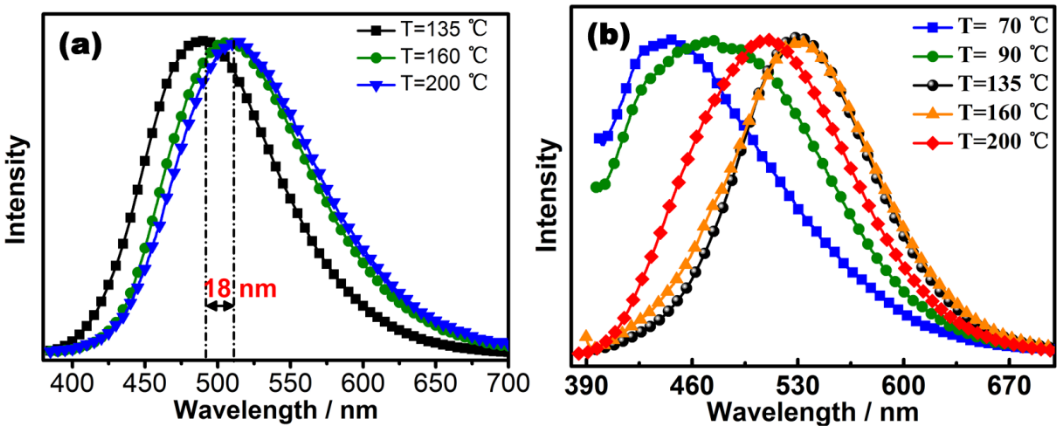

Figure S5. Effects of reaction temperature on PL properties of S2 (**a**) and S3 (**b**) at the same excitation wavelength.

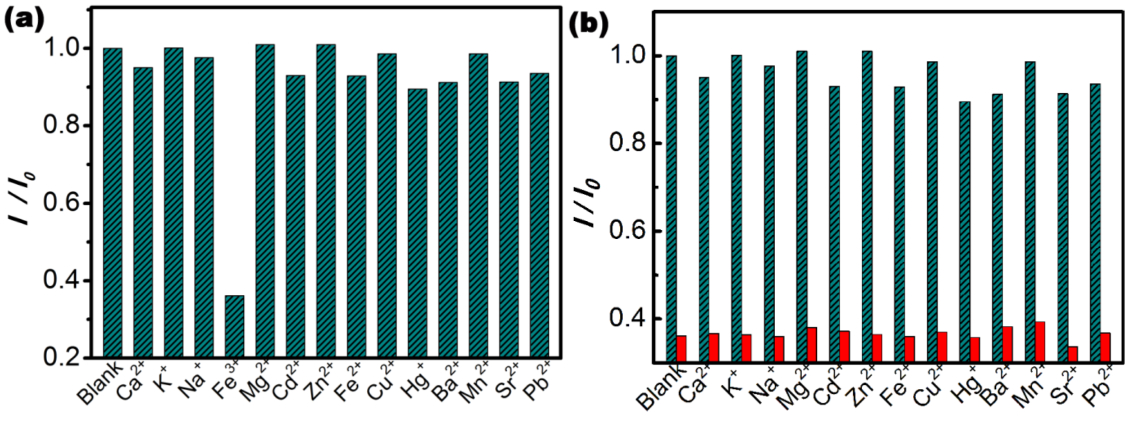

Figure S6. (**a**) PL intensity ratio (I/I_0) of the S2 in the absence and presence of different metal ions (1 mM); (**b**) The I/I_0 comparison of S2 in the presence of Fe^{3+} ions (1 mM) upon adding different metal ions (1 mM) listed from left to right: blank, Ca^{2+}, K^+, Na^+, Mg^{2+}, Cd^{2+}, Zn^{2+}, Fe^{2+}, Cu^{2+}, Hg^+, Ba^{2+}, Mn^{2+}, Sr^{2+}, and Pb^{2+}.
